# Is polysomnographic examination necessary for subjects with diaphragm pathologies?

**DOI:** 10.6061/clinics/2016(09)04

**Published:** 2016-09

**Authors:** Ozlem Oruc, Sema Sarac, Gulgun Cetintas Afsar, Ozgur Bilgin Topcuoglu, Serda Kanbur, Irfan Yalcinkaya, Fatma Merve Tepetam, Gokhan Kirbas

**Affiliations:** I Sureyyapasa Chest & Thoracic Surgery Training and Research Hospital, Chest Diseases, Istanbul, Turkey; II Sureyyapasa Chest & Thoracic Surgery Training and Research Hospital, Neurology, Istanbul, Turkey; III Sureyyapasa Chest & Thoracic Surgery Training and Research Hospital, Thoracic Surgery, Istanbul, Turkey; IV Sureyyapasa Chest & Thoracic Surgery Training and Research Hospital, Allergy and Immunology, Istanbul, Turkey; V Faculty of Medicine Dicle University, Chest Diseases, Diyarbakir, Turkey

**Keywords:** Obstructive Sleep Apnea Syndrome, Diaphragm Eventration, Diaphragm Paralysis, Diaphragmatic Plication

## Abstract

**OBJECTIVES:**

While respiratory distress is accepted as the only indication for diaphragmatic plication surgery, sleep disorders have been underestimated. In this study, we aimed to detect the sleep disorders that accompany diaphragm pathologies. Specifically, the association of obstructive sleep apnea syndrome with diaphragm eventration and diaphragm paralysis was evaluated.

**METHODS:**

This study was performed in Süreyyapasa Chest Diseases and Thoracic Surgery Training and Research Hospital between 2014–2016. All patients had symptoms of obstructive sleep apnea (snoring and/or cessation of breath during sleep and/or daytime sleepiness) and underwent diaphragmatic plication via video-assisted mini-thoracotomy. Additionally, all patients underwent pre- and postoperative full-night polysomnography. Pre- and postoperative clinical findings, polysomnography results, Epworth sleepiness scale scores and pulmonary function test results were compared.

**RESULTS:**

Twelve patients (7 males) with a mean age of 48 (range, 27-60) years and a mean body mass index of 25 (range, 20-30) kg/m2 were included in the study. Preoperative polysomnography showed obstructive sleep apnea syndrome in 9 of the 12 patients (75%), while 3 of the patients (25%) were regarded as normal. Postoperatively, patient complaints, apnea hypopnea indices, Epworth sleepiness scale scores and pulmonary function test results all demonstrated remarkable improvement.

**CONCLUSION:**

All patients suffering from diaphragm pathologies with symptoms should undergo polysomnography, and patients diagnosed with obstructive sleep apnea syndrome should be operated on. In this way, long-term comorbidities of sleep disorders may be prevented.

## INTRODUCTION

The diaphragm is the primary muscle of respiration 1. Paralysis and eventration of the diaphragm are uncommon conditions and can cause dyspnea 2-3. Unilateral or bilateral diaphragm paralysis following phrenic nerve injury may be caused by cardiac injury, trauma, mediastinal tumors, infections of the pleural space or motor neuron diseases. Eventration is a congenital defect, although eventration and paralysis have similar physiopathology 2-3. Because of the location of its points of insertion on the chest wall, caudal movement of the diaphragm in normal individuals during inspiration causes the rib cage to expand, which produces negative pleural pressure and lung inflation. In patients with diaphragmatic eventration or paralysis, caudal movement of the diaphragm is less effective (in eventration) or absent (in paralysis) 4. As a result, ventilation is impaired. Furthermore, perfusion to the basal portion of the lung ipsilateral to the paralyzed or eventrated diaphragm is also impaired, possibly owing to regional vasoconstriction induced by alveolar hypoxia. The resultant ventilation/perfusion mismatch and loss of pulmonary and chest wall compliance are among the factors that contribute to dyspnea 3,4. Diaphragmatic plication (DP) via video-assisted mini-thoracotomy (VAMT) is an effective, curative and minimally invasive technique used for the surgical correction of these pathologies 2,5,6,7,8,9,10,11,12,13.

Obstructive sleep apnea syndrome (OSAS) is a highly prevalent disease, affecting approximately 3%-10% of the adult population 14,15. It is typically characterized by recurrent upper airway (UA) collapse 16 and recurrent hypoxia 17. OSAS, which is known to affect almost all systems in the body, is associated with independent risk factors for coronary heart disease, including prevalent and incident hypertension, nocturnal nondipping of blood pressure, stroke and cognitive impairment 14-18.

Multiple pathophysiological risk factors contribute to anatomical abnormalities in OSAS, including elevated UA mechanical load, compromised UA dilators, increased loop gain, unstable respiratory control, and decreased arousal threshold 19. Sleep-related hypoxemia has been formerly described in patients with generalized muscle weakness as well as in those with bilateral diaphragm dysfunction (DD) 20. However, only a limited number of studies have assessed diaphragmatic activity during sleep, and these have had small sample sizes 20-22. Our study focused on the link between OSAS and unilateral DD and the effects of DP on OSAS. Notably, this is the first study to evaluate pre- and postoperative PSG results in this context.

The main goal of this study was to investigate the presence of OSAS in patients with symptomatic diaphragm paralysis or diaphragm eventration. We hypothesized that because of the similar pathogeneses of these two anatomical conditions and OSAS, they might accompany each other. Secondly, we compared pre- and postoperative PSG and pulmonary function test (PFT) results for patients with OSAS who underwent DP via VAMT to evaluate the effects of surgery on OSAS and pulmonary function. Knowing that unilateral DD may lead to sleep hypoventilation 20, we propose that patients with diaphragm pathologies may have a tendency for OSAS, which is a major cause of cerebro-cardio-vascular morbidity and mortality. With early diagnosis and treatment of OSAS, long-term co-morbidities in patients with diaphragm pathologies can be prevented.

## METHODS

### Patients

This study was performed in Süreyyapaşa Chest Diseases and Thoracic Surgery Training and Research Hospital, İstanbul, between January 2014 and January 2016. Twenty-two patients with diaphragm paralysis and/or eventration who were candidates for DP were examined in the Sleep Laboratory of our hospital and questioned about OSAS symptoms. Fourteen patients complained about snoring and/or cessation of breath during sleep and/or daytime sleepiness. Two patients dropped out of the study before it started for personal reasons. Therefore, twelve patients were included in the study. The demographic variables (age, gender, and body mass index (BMI)) of the patients were collected. BMI was calculated as the ratio of dry weight in kilograms to height in meters squared. Daytime sleepiness was evaluated with the Epworth Sleepiness Scale (ESS). The patients underwent PSG and PFTs one month before and one month after the operation. Pre- and postoperative PSG and PFT results were compared.

### Pulmonary Function Test

All patients underwent PFTs using a ZAN 74N (2007, USA) device. The same technician performed all PFTs, which were conducted at least three times per patient per test session in the sitting position. Forced expiratory volume in the first second (FEV1) and forced vital capacity (FVC) values were recorded.

### Polysomnography

All patients underwent a nocturnal PSG, which was performed with multichannel monitoring that included neurophysiological electrodes (electroencephalography electrodes), chest wall motion, abdominal motion, arterial oxygen saturation and electrocardiography electrodes (Grass-Telefactor Cephalo, An Astro-med Inc. Product Group, 2005, USA). Oronasal airflow was measured by a thermistor. Oxyhemoglobin saturation was monitored with a finger pulse oximeter with a sampling rate of 1 Hz. Body position was measured by a position sensor attached to the anterior chest wall. Signals recorded in the sleep period were manually analyzed 23. Scorings were made by certified physicians experienced in sleep medicine. Apnea was scored as a decrease in airflow of at least 90% from baseline for at least 10 seconds and classified as central, mixed or obstructive depending on the presence of thoracoabdominal movements 23. Hypopnea was scored as a decrease in airflow of at least 30% for ≥10 seconds accompanied by a SaO2 (oxygen saturation) decrease ≥3%. Apnea/hypopnea index (AHI) was calculated as the average number of apneas and hypopneas per hour of sleep 23. SaO2 during sleep was automatically recorded: the mean SaO2 and lowest nocturnal SaO2 values were detected. Patients with an AHI≥5 were considered to have OSAS. As recommended by the American Academy of Sleep Medicine task force, the severity of sleep apnea was classified as mild if the AHI ranged from 5 to 15/h, moderate when the range was 15 to 30/h, and severe when the AHI was >30/h 23. Patients were classified as having supine-related OSA on the basis of the following two definitions: 1 supine-predominant OSA, in which the supine AHI to non-supine AHI ratio is ≥2 to 1 and 2 supine-isolated OSA, in which the supine AHI to non-supine AHI ratio is ≥2 to 1 and the non-supine AHI is <5 events/hour 24-25.

### Statistical analysis

Data were analyzed with SPSS statistical software version 20 (Chicago, Illinois, USA). Descriptive statistics were described as frequencies, percentages, mean values +/-standard deviations (SDs) or median values (min-max). For normally distributed continuous variables, a paired Studentߣs t test was used, and for abnormally distributed variables, a paired Wilcoxon signed-rank test was used. Statistical significance was set at *p*<0.05.

### Ethics

This study was approved by the Local Ethics Committee of our institution and was conducted in accordance with the ethical principles stated in the Declaration of Helsinki. Written informed consent was obtained from each participant included in the study.

## RESULTS

Twelve patients (7 males, 5 females) underwent DP via VAMT (8 left-sided and 4 right-sided). The etiology was undefined in 8 patients (66.7%), whereas 2 patients (16.7%) had previous surgery and 2 (16.7%) had trauma history. Eight patients (66.7%) complained about respiratory problems (2 patients had dyspnea with chest pain, 8 patients had only dyspnea), and 4 patients (33.3%) had combined respiratory and gastrointestinal symptoms (bloating and abdominal pain after meals). When the patients were asked about OSAS symptoms, 9 patients complained about only snoring and 3 patients complained about snoring and cessation of breath during sleep. None of the patients had daytime sleepiness. The mean age of the patients was 48 years (range, 27 to 60 years) and the mean BMI was 25 kg/m^2^ (range, 20-30).

Nine patients (75%) were diagnosed with OSAS based on preoperative PSG. Regarding supine and non-supine AHIs, 8 of the patients (88.9%) had supine-related OSAS. Regarding overall AHIs, 3 patients (33.3%) had severe OSAS, 4 patients (44.4%) had moderate OSAS and 2 patients (22.2%) had mild OSAS. Postoperative PSG was performed 1 month after surgery, and all 9 patients showed improvement, with complete recovery (overall AHI<5) being achieved in 2 patients (22.2%) and decreased OSAS severity being achieved in 7 patients (77.8%). The mean ESS also decreased from 4.67 to 2.75.

The pre- and postoperative AHIs, diagnoses and preferred types of treatment for the patients are summarized in Table 1.

Pre- and postoperative PSG results, ESS scores and PFT results were compared. Total sleep time (TST), sleep efficiency and minimum SPO2 were significantly higher postoperatively (*p*=0.029, *p*=0.005, and *p*=0.048, respectively), while overall AHI, supine AHI, ODI and ESS scores were significantly lower (*p*=0.004, *p*=0.001, *p*=0.01, and *p*=0.001, respectively). Additionally, significant improvements in FEV1 (ml), FEV1 (%) and FVC (%) (*p*=0.024, *p*=0.001 and *p*=0.003, respectively) were found postoperatively (Table 2).

## DISCUSSION

This study showed that OSAS is very common (75%) in patients with unilateral diaphragm pathologies compared to the normal population. Furthermore, DP via VAMT resulted in improvements in both subjective and objective PSG and PFT results.

Previous studies on sleep disorders in unilateral or bilateral diaphragm paralysis have produced conflicting results 26-33. While some studies have reported DDs to cause disturbed sleep, inadequate ventilation during sleep and daytime sleepiness 26,30, others have found insignificant changes in normal sleep pattern unless there is additional load on the ventilatory system 28,29,31. Baltzan reported that individuals with isolated unilateral diaphragm diseases had position-dependent hypopneas during the REM period of sleep with frequent desaturations; therefore, unilateral diaphragm paralysis might be a significant and underdiagnosed cause of sleep disordered breathing in these patients 22. Our results showed that disturbed sleep without daytime sleepiness and position dependency were detected in 88.9% of our patients, and these factors were unrelated to REM sleep.

REM stage sleep in young adults consists of 20%-25% of TST 34. Preoperative PSGs of our patients showed lower percentages of REM sleep, indicating sleep disturbance. However, the postoperative PSGs, consistent with the literature, showed enhancement of REM sleep and TST with increased sleep efficiency 20,21,22, demonstrating an improvement in sleep quality. The observed improvements in the PFT results were also consistent with former literature 4,7,8,9,10,11.

Sleep position has a significant effect on respiratory mechanics. UA anatomy can predispose an individual to reductions in muscle tone. In the supine position, retroglossal narrowing of the UA is expected to increase significantly in patients who are affected. Regional vasoconstriction, which impairs perfusion to the basal portion of the lung due to alveolar hypoxia, increases airway resistance. In the supine position, the contribution of chest-wall expansion does not exceed the effect of increased abdominal distention, and functional residual capacity is reduced. With REM sleep-associated atonia, skeletal muscles responsible for respiration are significantly impaired and ventilation is achieved by the diaphragm alone. Chest-wall compliance is increased with this decreased intercostal tone, and paradoxical collapse of the chest during inspiration may occur 35. Diaphragmatic contraction expands intrathoracic volume by displacing the abdominal viscera and expanding the rib cage using the abdomen as a support. The resulting negative intrathoracic pressure induces a net influx of air into the lungs. When the diaphragm is paralyzed, work is generated entirely by the accessory muscles of respiration and the affected diaphragm remains stable or, worse, moves paradoxically with respiration. Paradoxical superior movement results in a greatly reduced change in intrathoracic volume with inspiration, leading to a more severe respiratory impairment than when the diaphragm remains in a fixed position. As the abdominal contents apply pressure on the diaphragm in the supine position, respiratory imbalances are often exacerbated. The clinical presentation of DD changes with the degree of paralysis, if the paralysis is unilateral or bilateral and whether an underlying pulmonary disease is present 1, but patients with diaphragm paralysis and eventration are usually asymptomatic 2,3. Dyspnea and orthopnea are the only indications for surgical treatment 2,3,4. The primary goal of DP for hemi-diaphragmatic paralysis or eventration is to relieve the symptom of dyspnea. Former studies have shown that DP can improve functional status, shortness of breath and PFT results. Surgical plication serves to prevent the paradoxical movement of abdominal organs to the ipsilateral thoracic cavity during inspiration by stabilizing the atrophic, thin, loose and elevated diaphragm, thus preventing any parenchymal atelectasis or inadequate shunting. Thus, diaphragmatic redundancy is reduced with plication, intrathoracic negative pressure for expansion of the ipsilateral lung is re-established, and exercise performance and tidal volume are increased 4,7,8,9,10,11,12,13.

The relationships between sleep apnea and cerebro-cardio-vascular co-morbidities such as hypertension, heart failure, coronary artery disease, stroke and sudden death are well known 14,15,16,17,18,34. It is important to assess postoperative decreases in AHI and ODI values. Hence, we propose that all patients with diaphragm paralysis or eventration should undergo PSG and that sleep disorders should be considered as an indication for operation even in the absence of dyspnea and orthopnea. In this way, long-term complications of OSAS may also be prevented.

OSAS is frequent in patients with diaphragm pathologies. Plication of the hemi-diaphragm using minimally invasive techniques produces significant improvements in symptoms and pulmonary spirometry and PSG results. All symptomatic patients with diaphragm pathologies should undergo PSG.

## AUTHOR CONTRIBUTIONS

Oruc O was responsible for study design, data analysis and manuscript preparation. Sarac S and Afsar GC were responsible for the literature search and data collection. Topcuoglu OB was responsible for data collection, study design and manuscript preparation. Kanbur S was responsible for the literature search and analysis of data. Yalcinkaya I was responsible for study design and manuscript review. Tepetam FM was responsible for study design and manuscript preparation. Kirbas G was responsible for data analysis and manuscript review.

## Figures and Tables

**Table 1:** Preoperative and postoperative PSG results and the preferred types of treatment.

	Preop AHI	Preop NS AHI	Preop S AHI	Preop ODI	Preop diagnosis	Postop AHI	Postop NS AHI	Postop S AHI	Postop ODI	Postop diagnosis	Treatment
#1	22.5	0.8	62.5	19.3	Supine-isolated ModerateOSAS	22.8	2.9	48.6	22.4	Supine-isolated Moderate OSAS	Auto CPAP
#2	43.6	11.8	60.2	34.6	Supine-predominant Severe OSAS	12.5	6.8	18.7	11.2	Supine-predominantMild OSAS	Follow up
#3	25.4	8.3	40.1	24.0	Supine predominantModerateOSAS	10.5	11.6	6.8	14.1	Non-supine relatedMild OSAS	Follow up
#4	57.5	14.9	83.7	40.9	Supine predominantSevere OSAS	26.5	17.8	41.5	14.2	Supine predominant Moderate OSAS	Auto CPAP
#5	36.8	28	62.3	37.6	Supine predominantSevere OSAS	14.8	5	21.8	18.1	Supine isolatedMild OSAS	Follow up
#6	22.6	13.0	30.0	19.7	Supine predominantModerateOSAS	12	3.2	22.9	11.9	Supine isolatedMild OSAS	Follow up
#7	7.4	7.6	7.4	3.0	Non-supine related Mild OSAS	1.2	0.8	1.4	1.05	Normal	
#8	19.8	11.1	44.2	19.0	Supine predominantModerate OSAS	7.1	2.8	9.1	6.3	Supine isolatedMild OSAS	Follow up
#9	5.3	1.7	11.2	4.6	Supine isolated Mild OSAS	1.3	0.7	1.9	2.5	Normal	
#10	4.1	0.00	10.8	4.0	Normal						
#11	1.3	0.5	1.7	1.3	Normal						
#12	4.2	1.5	7.8	3.8	Normal						

S AHI: supine AHI, NS AHI: non-supine AHI, CPAP: continuous positive airway pressure.

OSAS: obstructive sleep apnea syndrome, ODI: oxygen desaturation index.

Preop: preoperative, postop: postoperative.

**Table 2:** Preoperative and postoperative PSG and PFT results.

	Preoperative	Postoperative	*p* value
Total sleep time (TST)	373	412	**0.029**
Sleep efficiency	78.89	89.83	**0.005**
AHI	26.77	12	**0.004**
Non-supine AHI	10.80	5.89	0.12
Supine AHI	44.62	19.33	**0.001**
NREM stage 1 (%)	7.03	2.59	**0.013**
NREM stage 2 (%)	49.66	63.4	**0.007**
NREM stage 3 (%)	23.38	14	0.21
REM stage (%)	13.18	19.4	0.35
Minimum SPO2	79	86	**0.048**
ODI	22.52	11.11	**0.01**
ESS	4.67	2.75	**0.001**
FEV1	2.09	2.25	**0.024**
FEV 1 (%)	66.92	74.67	**0.001**
FVC	2.555	2.750	0.263
FVC (%)	65.67	76.33	**0.003**

ODI: oxygen desaturation indices, ESS: Epworth Sleepiness Scale, TST: total sleep time, FEV1: forced expiratory volume in the first second, FVC: forced vital capacity.
